# Unwanted and Illegitimate

**Published:** 1920-03-13

**Authors:** 


**THE PUBLIC WELL-BEING—(*continued*).**

### Unwanted and Illegitimate.

### Problem Considered from Two Angles.

Two women notable for good social work—Mrs. Lloyd George and Mrs. H. A. L. Fisher—have been responsible for bringing before the public recently two particular points of view in relation to the care of infants—that of the so-called "unwanted" babies, and that of the illegitimate child, both of them hard cases deserving sympathetic and practical consideration. The days are now past when irresponsible and reckless parents who take upon themselves so lightly the creation of precious life are to be damned through their unfortunate offspring. Everything should be done, it is agreed, for the infants, in order that they should have as fair a chance as possible, and towards the mothers the attitude of smug, harsh morality which treats them as outcasts to be unhelped and in fact penalised, has given place in all enlightened minds to more humane and more useful social considerations.

The problem of the "unwanted" baby was the point concerned in an "At Home" held in Downing Street by Mrs. Lloyd George in support of the National Children Adoption Association, whose prime object is to bring the homeless child to the childless home. It was explained that this was done at the offices in 19 Sloane Street, but it had been found essential in connection with the work to have a hostel, and one was established at Tower Cressy, Campden Hill. The work there began in July last, and in December it was formally opened by the patron, Princess Alice, who takes so much interest in child welfare. This hostel possesses the inestimable advantage of being able to say to would-be adopters: "Come and choose one." Through its doors have already passed nearly 100 babies into happy houses, where they have equal chances and opportunities with other children. Since the Association started it has had to deal with at least 6,000 cases, and it has placed in real homes at least 600 babies, and done it with an expenditure of about £3,000, working out at a cost of just £5 per child. If with one small organisation and one hostel could be found homes for ten babies a week, what could not be done if this work were developed all over the country, and not only in this country, but also in the Dominions; for inquiries have come from Australia, and much interest has been shown in the United States.

The other part, which is often the same part of the question as the one just mentioned, is outlined in articles in the *Observer* and *Daily News*, written by Mrs. Fisher, who is Chairman of the National Council for the Better Care of the Unmarried Mother and her Child. A Bill is to be introduced in Parliament by Mr. Neville Chamberlain, with the support of the main bodies concerned with this problem—bodies such as the National Council itself, for which Mrs. Fisher speaks, the N.S.P.C.C., and the Salvation Army. The Bill is based upon the principle of increasing and recognising the responsibility of the father, and, failing him, of the State, for the maintenance of the children. Illegitimate children are to be wards of court, and the duty of getting into touch with the father is placed upon an official of the court. The 10s. limit under affiliation orders is to be abolished, and the sum payable is to be arranged in accordance with the means of both parents and the real needs of the child. These are valuable proposals and should have the support of all who have realised for long that nothing like a proper attempt has been made by law to insist upon the father bearing his proper share of responsibility. To the objection that the increase of the father's responsibility and

**THE PUBLIC WELL-BEING**—(*continued*).

the lessening of the burden on the unmarried mother would tend to increase illegitimacy—an unmoral discrimination in favour of the father—Mrs. Fisher produces evidence against it from the experience of countries which have adopted legislation along the lines proposed. There the experience has been that, instead of a steady increase, there has been a steady decrease in the percentage of illegitimate births.

It is essential not only in the interests of the child, or even in the interests only of the mother, but also in the interests of the State, that the father should help to bear not only the moral, but also the economic, consequences of the parenthood of illegitimates. The infantile death-rate is at least twice that of the legitimate, and those who survive very often are brought up in circumstances which tend to do irretrievable damage to their future prospects. There is also this other point, that the greatest chance of helping the mother to regain her emotional balance and self-respect is to be found in the satisfaction of caring for her own child. Many people are working on this problem, which is being approached in a new spirit of enlightenment; but there is much yet to do, and the Bill which Mrs. Fisher outlines, at any rate, makes a start at a point which has been far too long neglected. Anyone with the smallest acquaintance with the courts knows how ridiculously disproportionate is the burden on the father. More often than not it is no burden at all.

